# Supplementary material for: Overnutrition in mice impairs thyroid hormone biosynthesis and utilization, causing hypothyroidism, despite remarkable thyroidal adaptations
Source: J Clin Invest. 2026 Apr 15;136(8):e194207. doi: 10.1172/JCI194207 (PMC13078877; doi:10.1172/JCI194207)

Figure 5E: BiP & Supplemental Figure 8A: Total protein

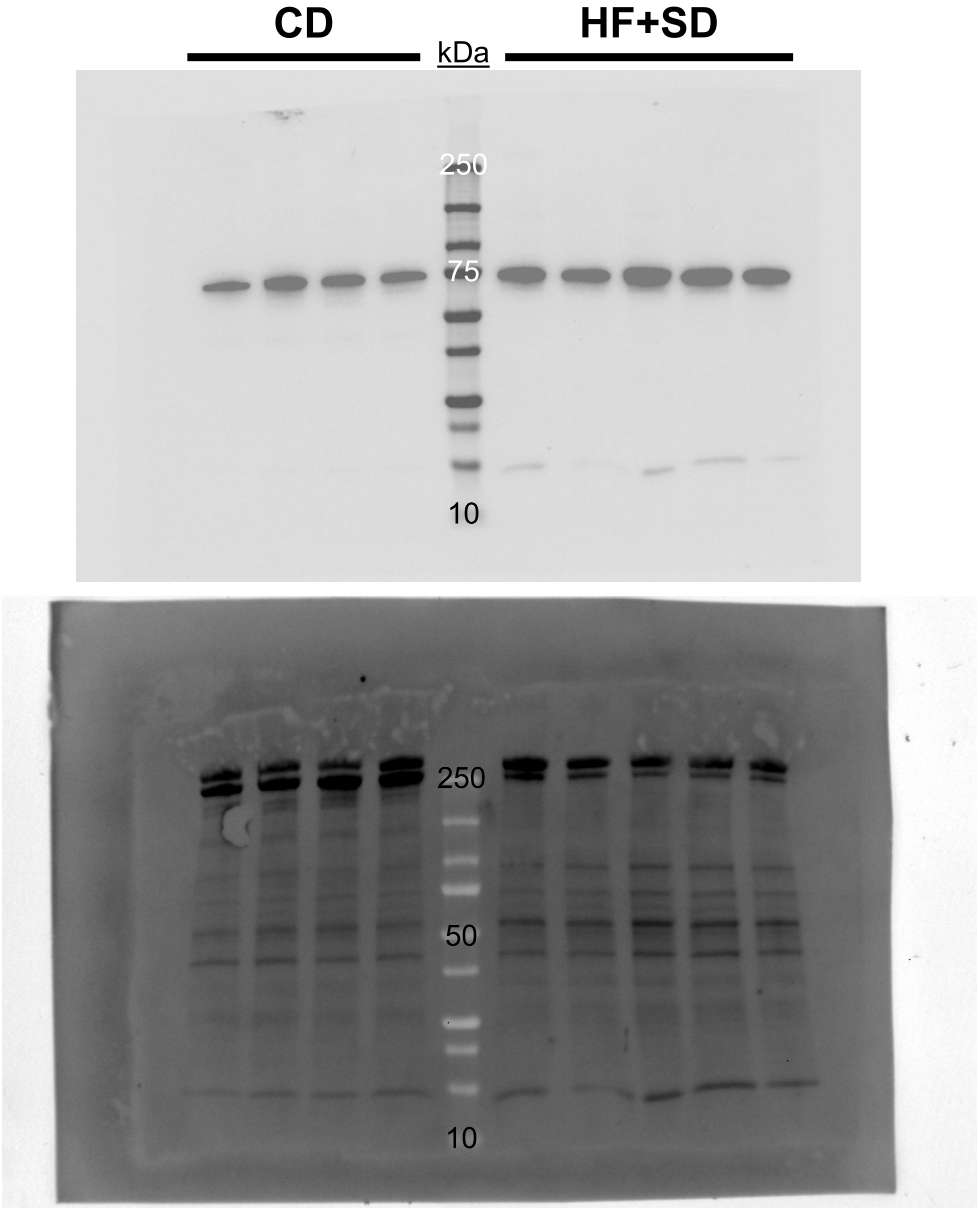

Figure 5E: p-eIF2α & t-eIF2α

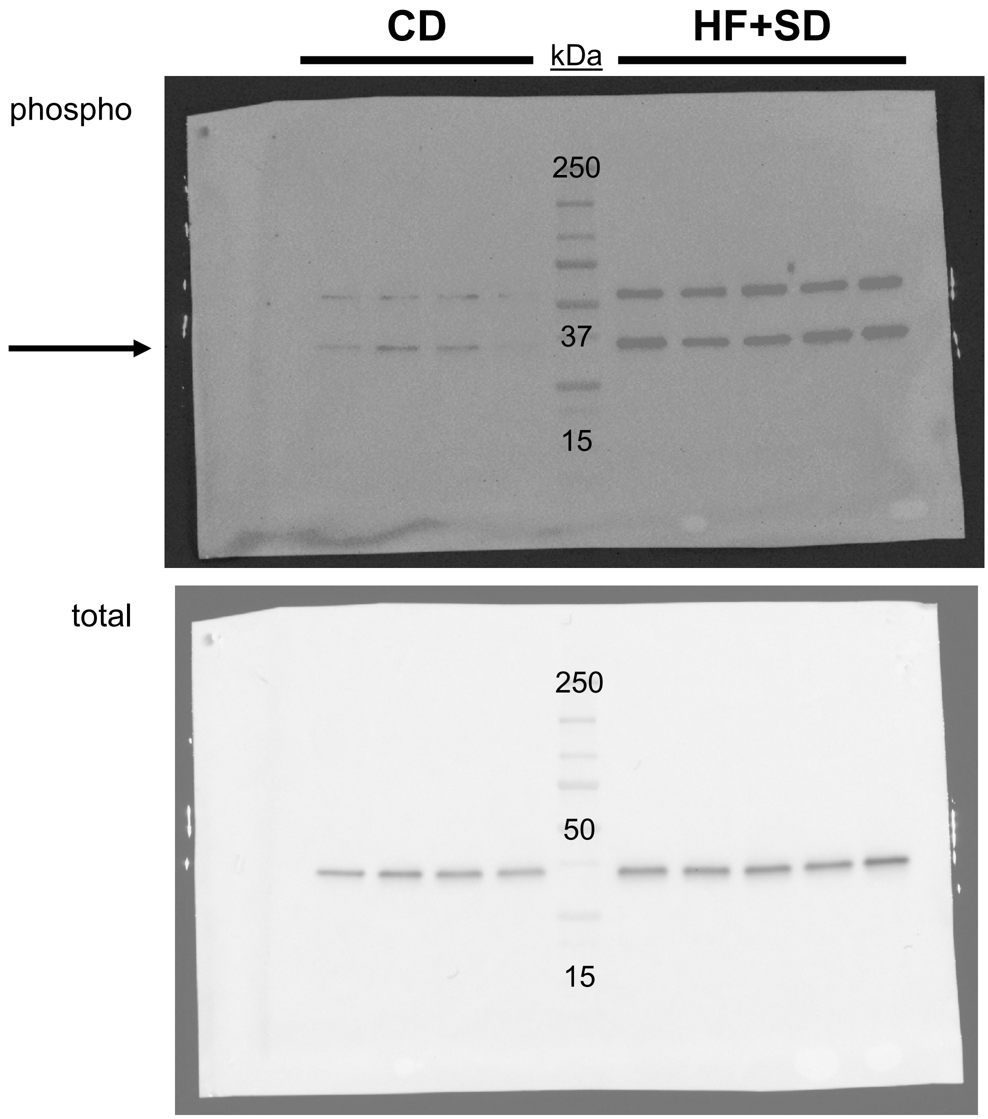

Figure 5E: CHOP & Supplemental Figure 8A: Total protein

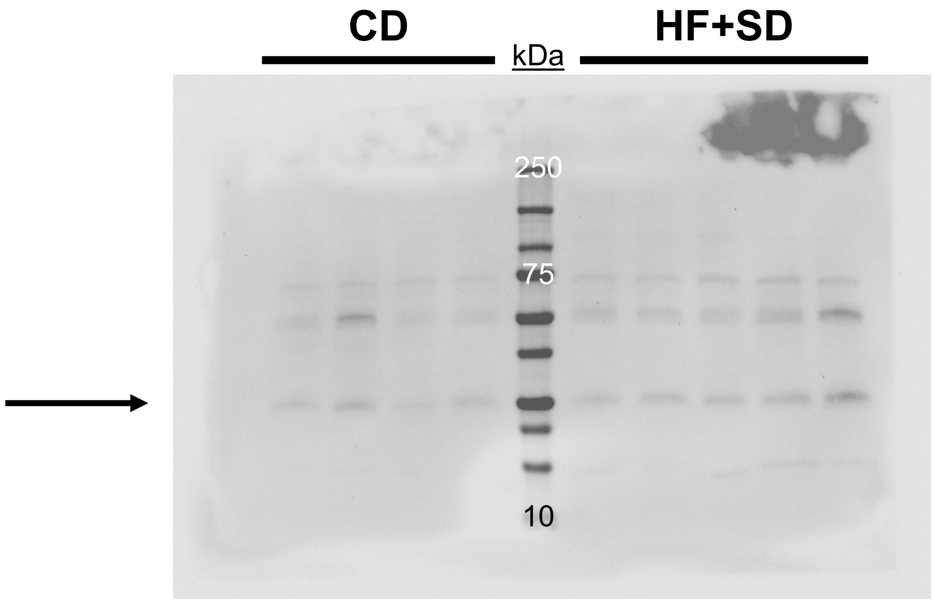

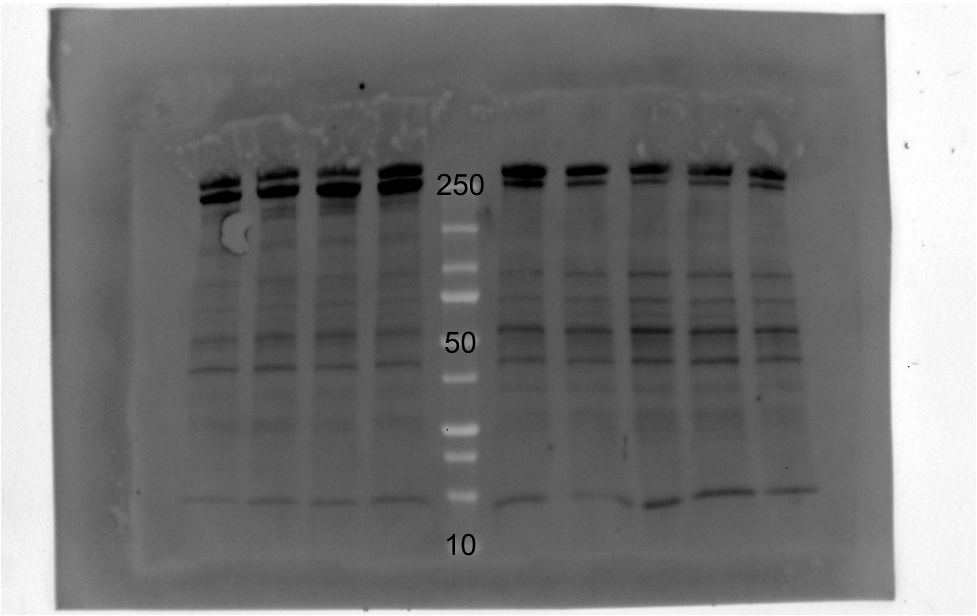

Figure 5E: TG & Supplemental Figure 8B: Total protein

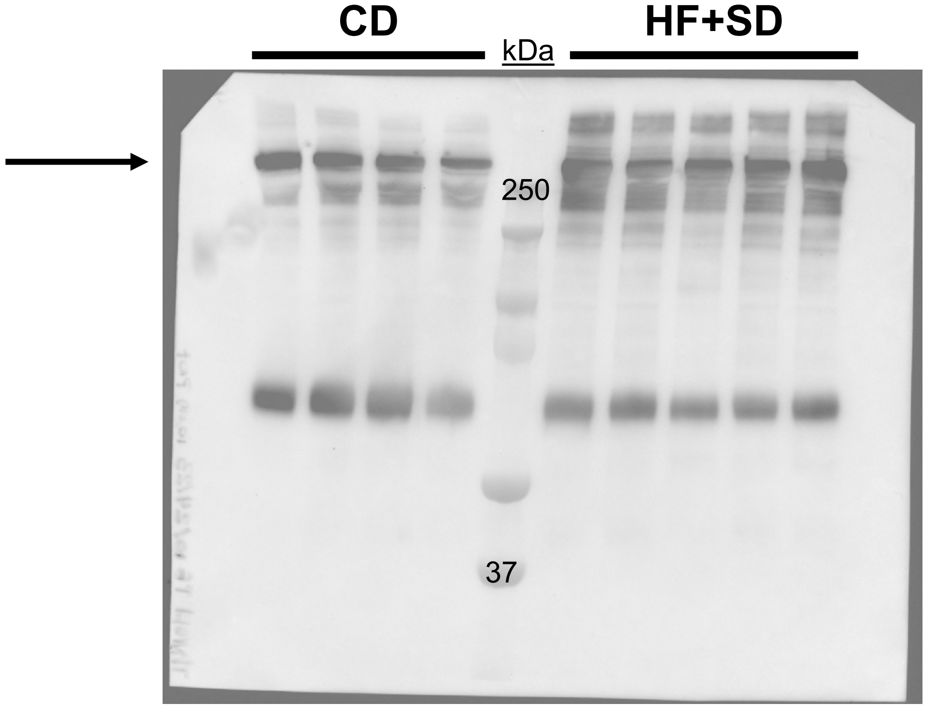

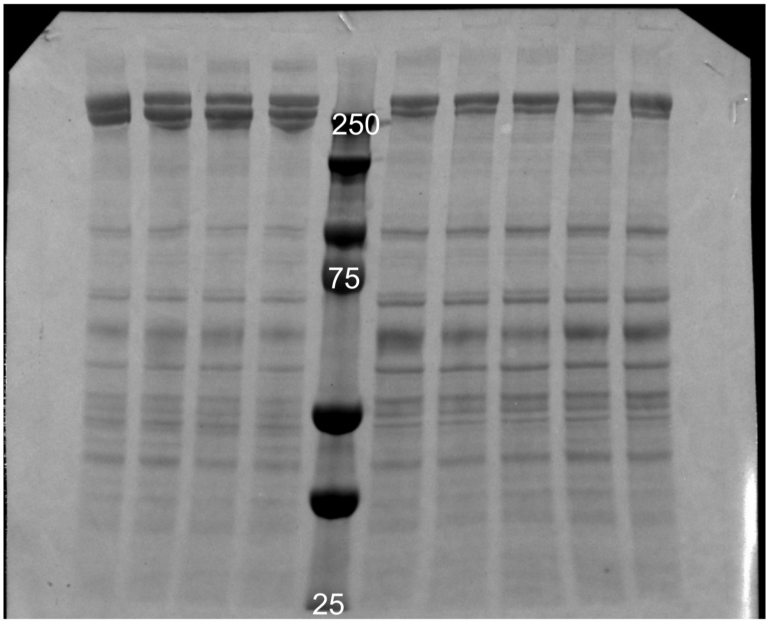

Figure 5H: PDI & vinculin

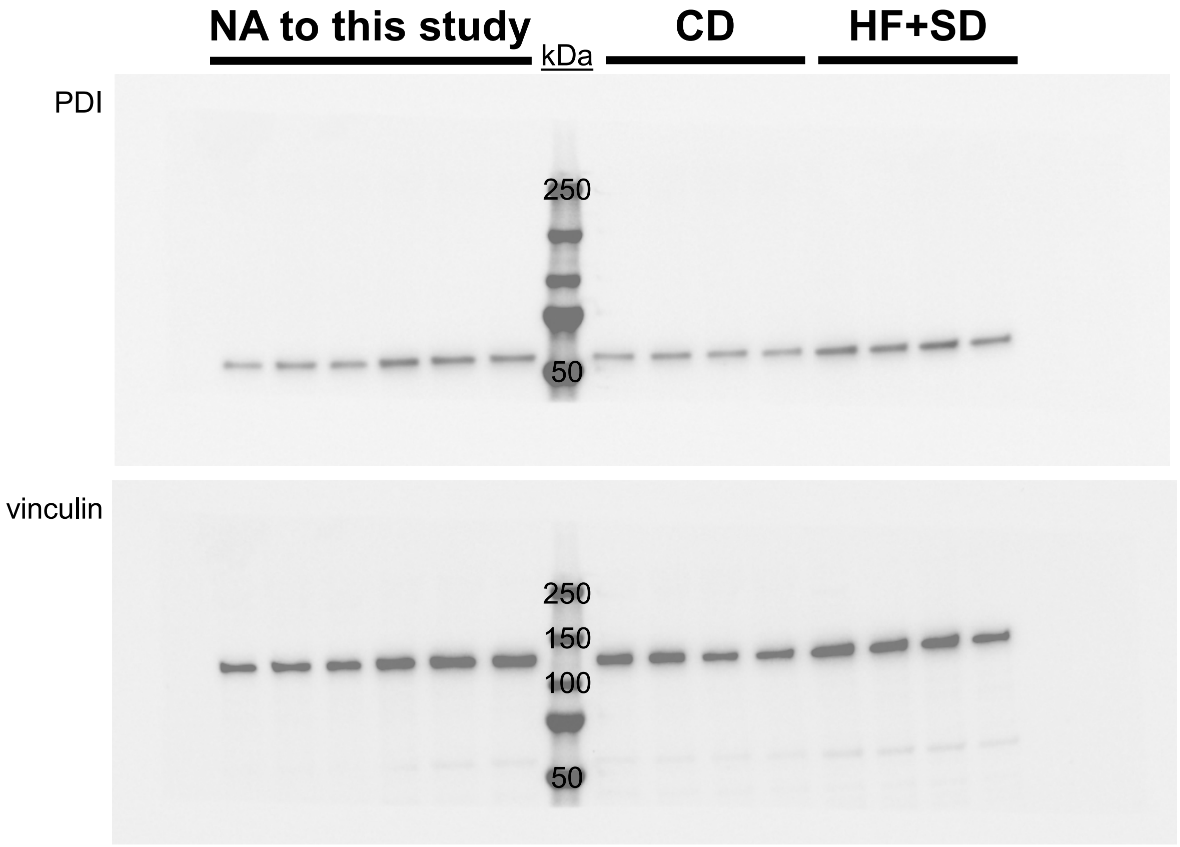

Figure 5H: Ero1-Lα & vinculin

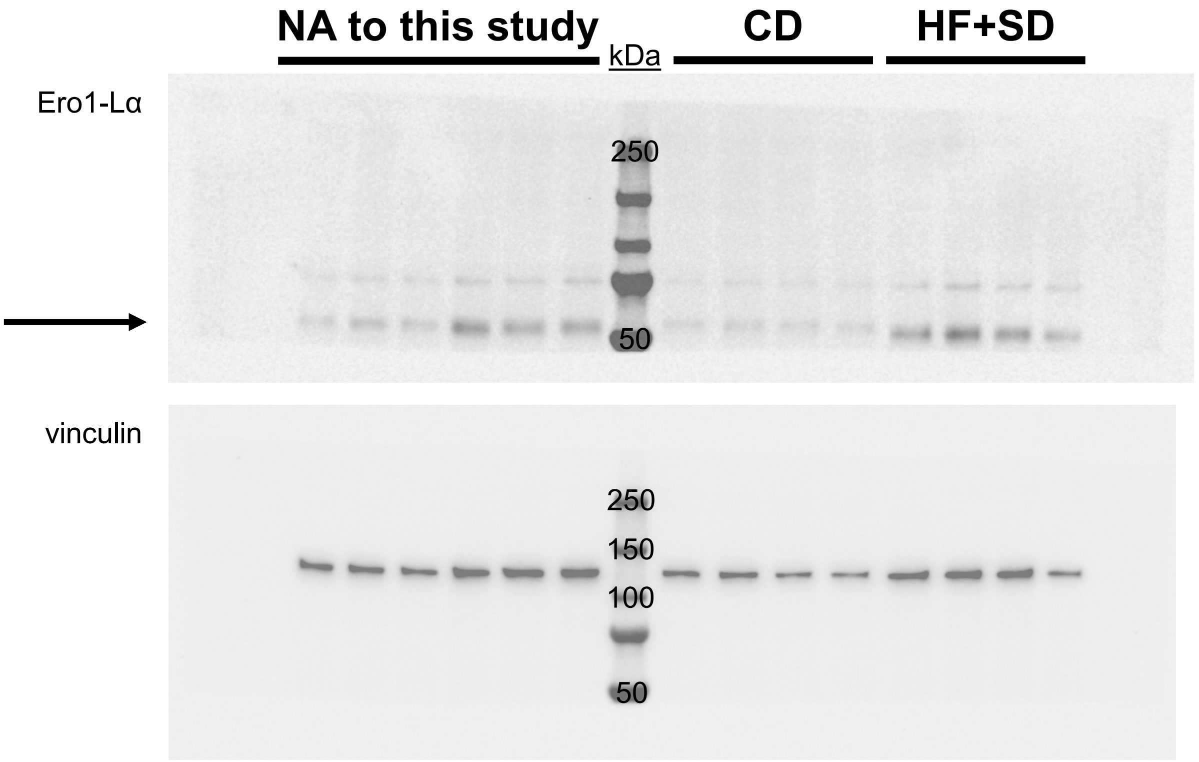

Figure 6B: NIS & Supplemental Figure 10A: Total protein

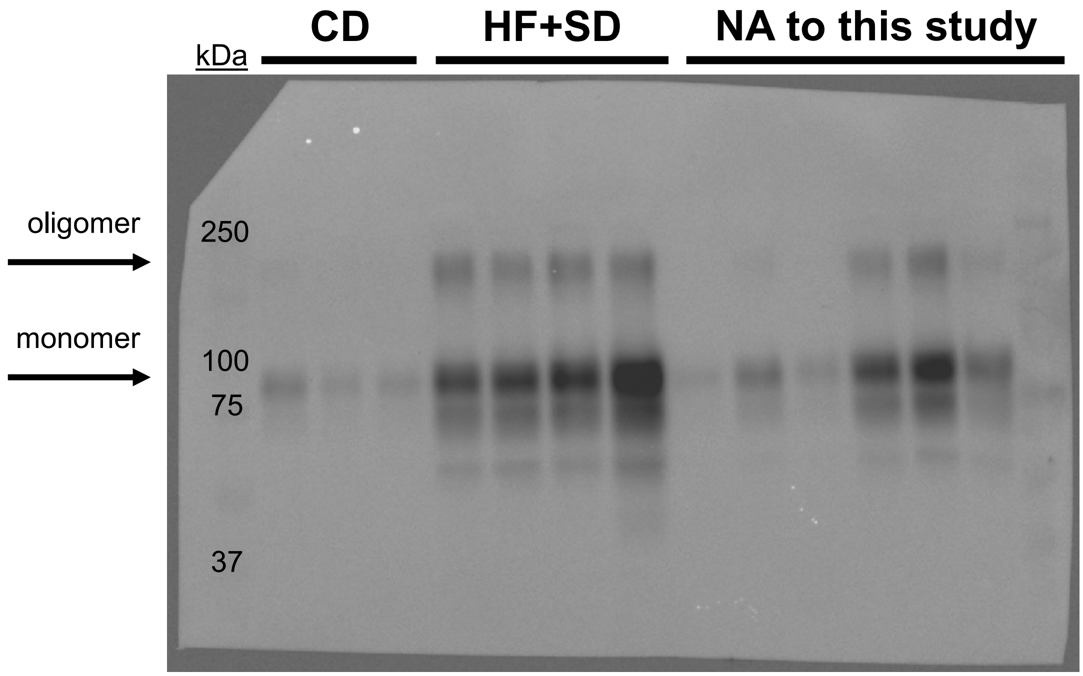

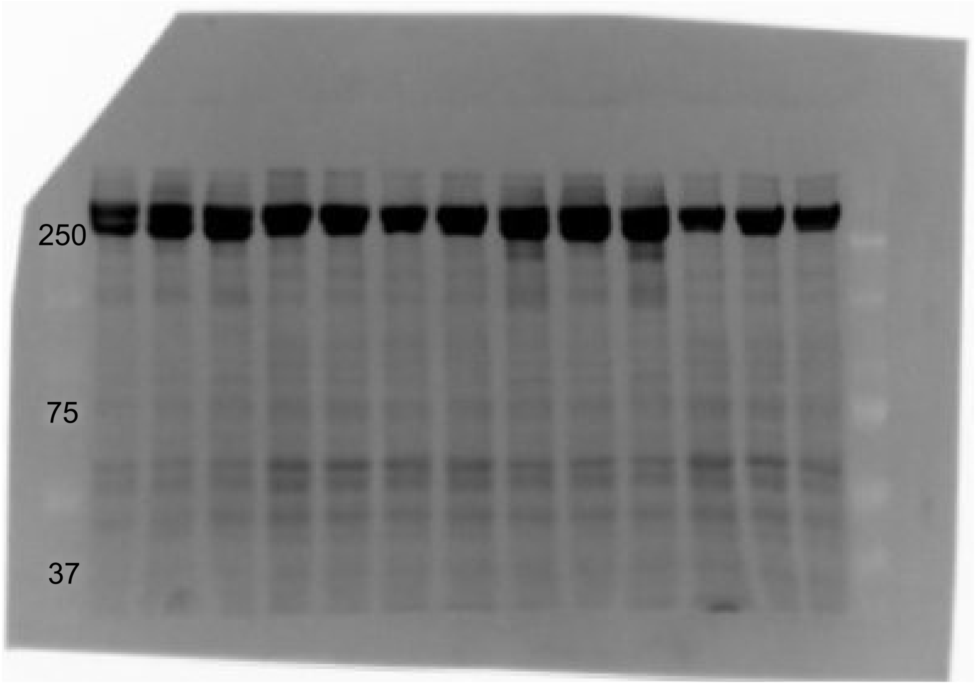

Figure 7J: TG & β-actin

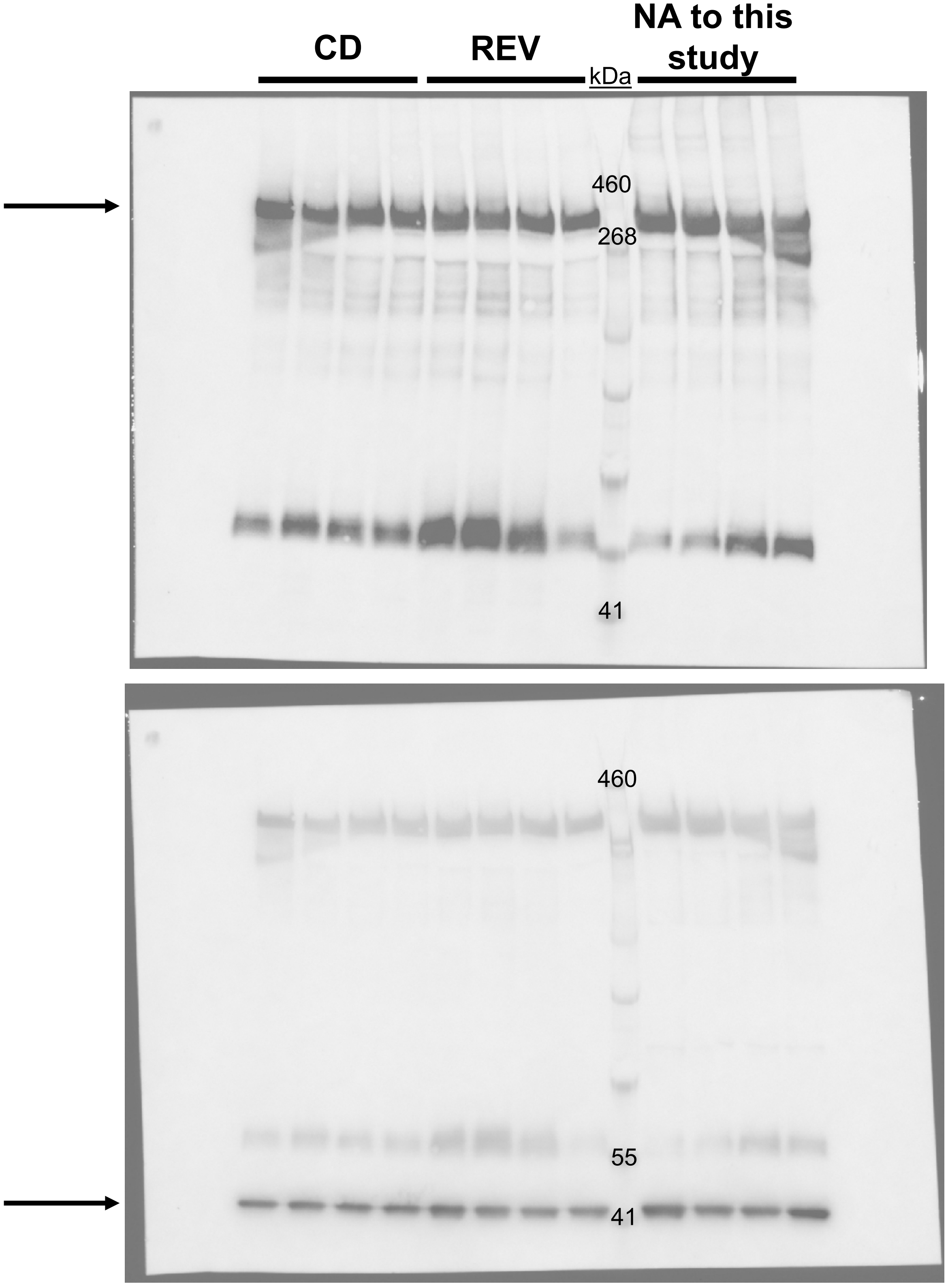

Supplemental Figure 3A: D2 & cav-1

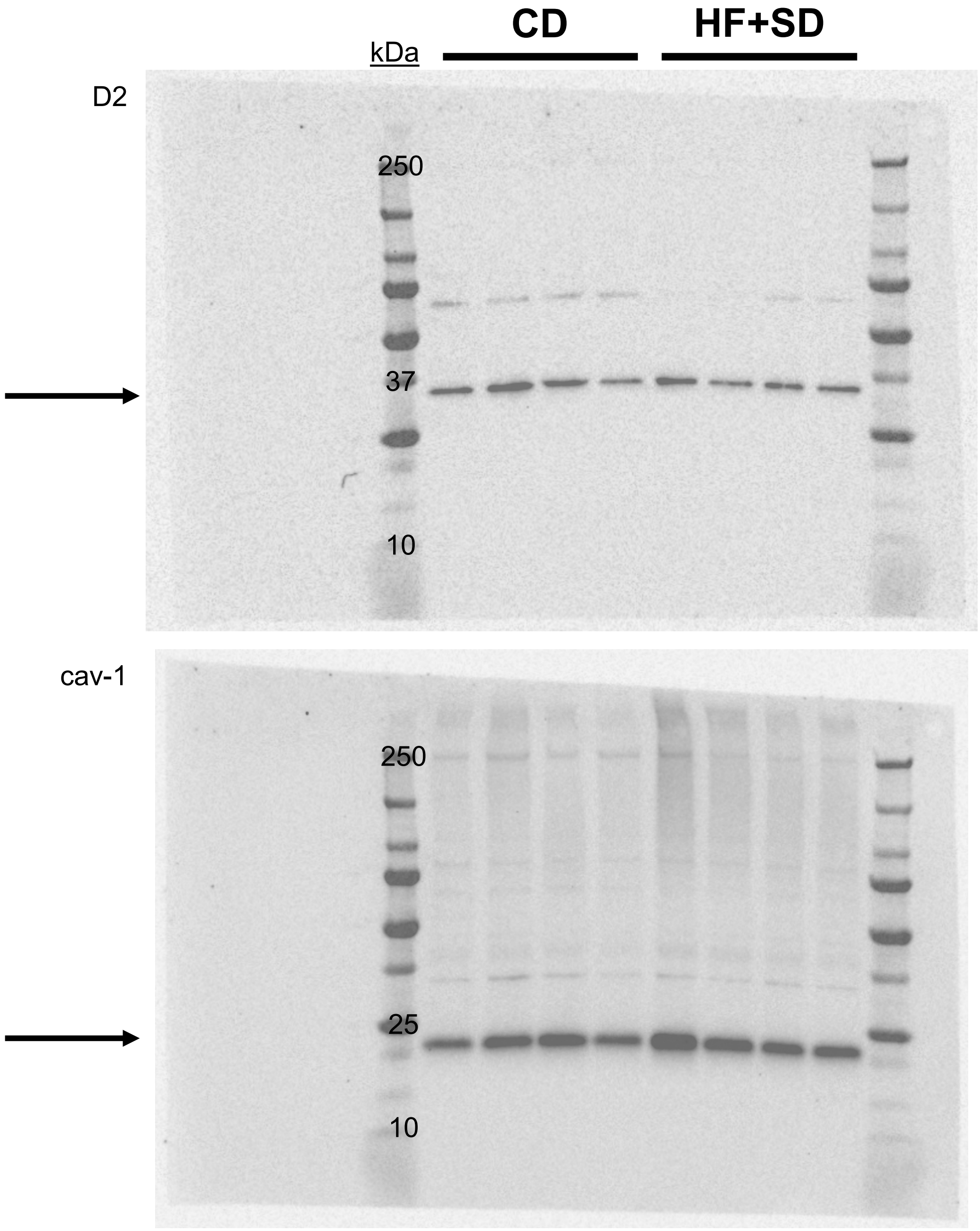

Supplemental Figure 7A: OxPhos & total protein

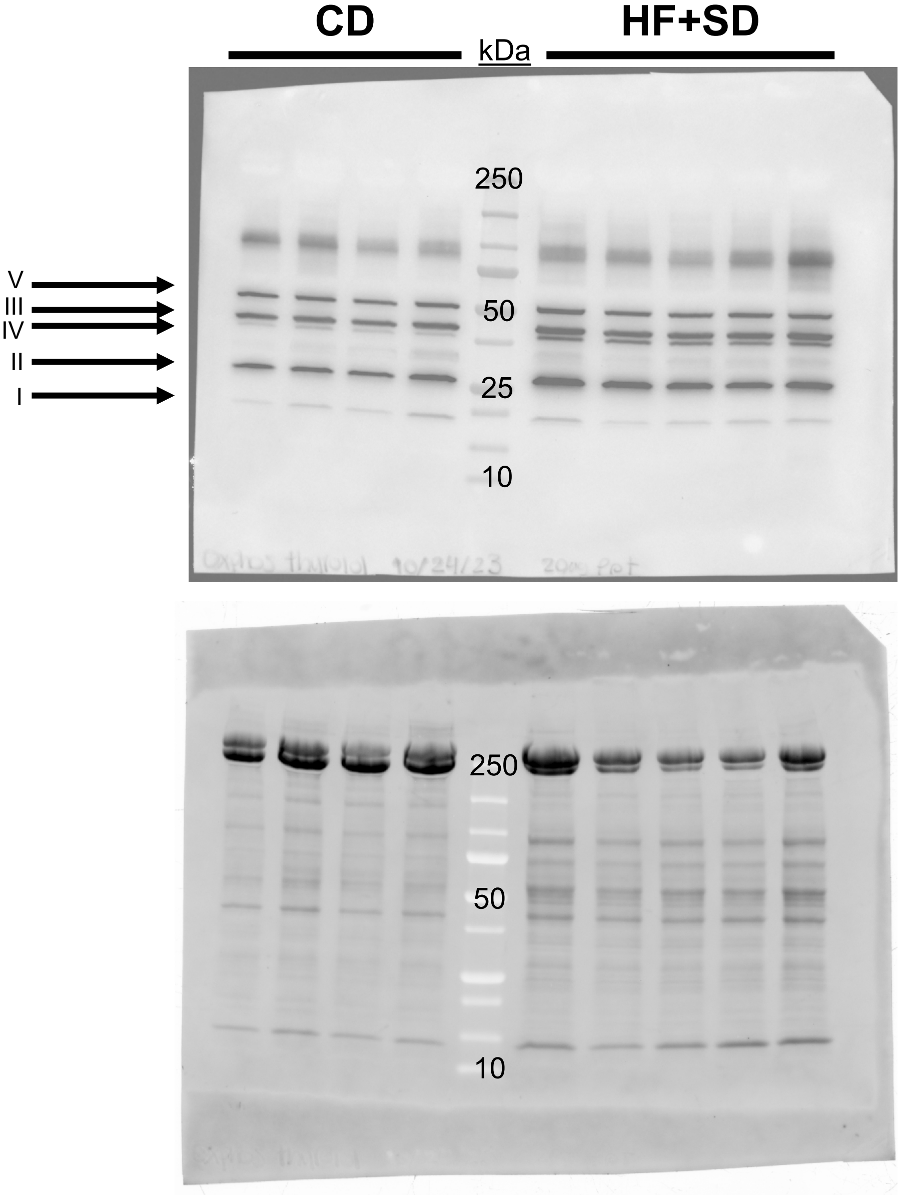

Supplemental Figure 10B: NIS & total protein

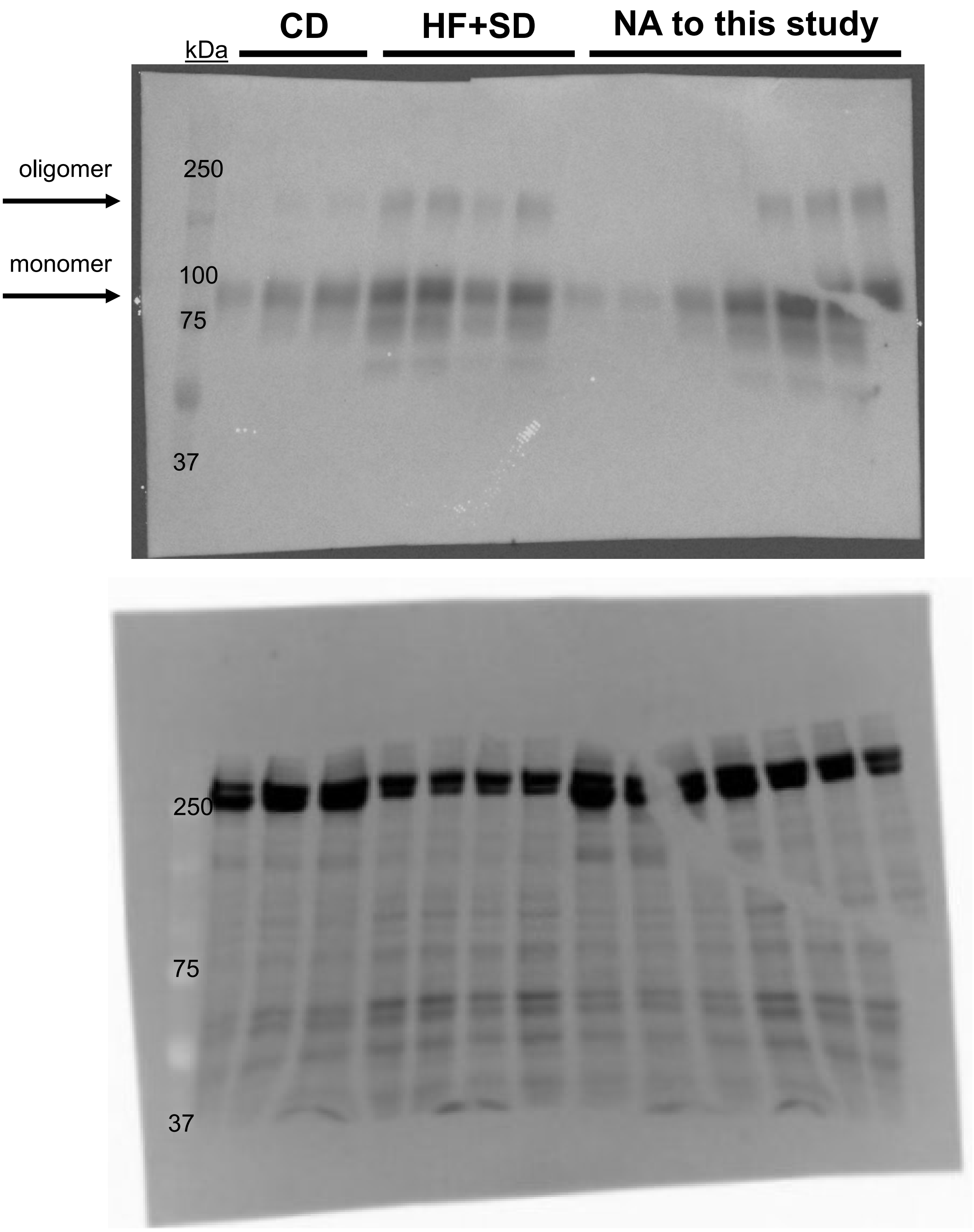

Supplemental Figure 12A: BiP & total protein

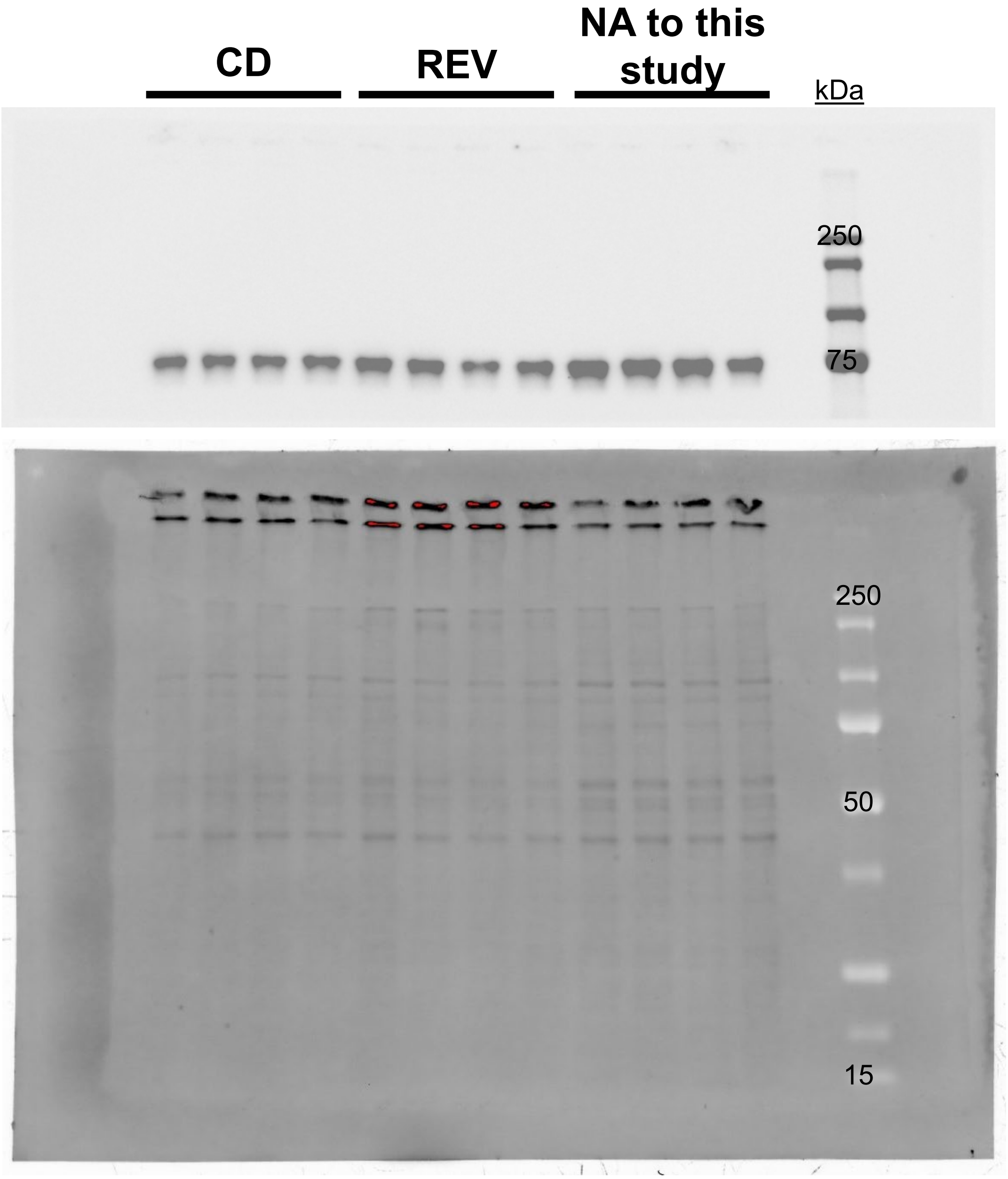

Supplemental Figure 12B: p-eIF2α & total protein

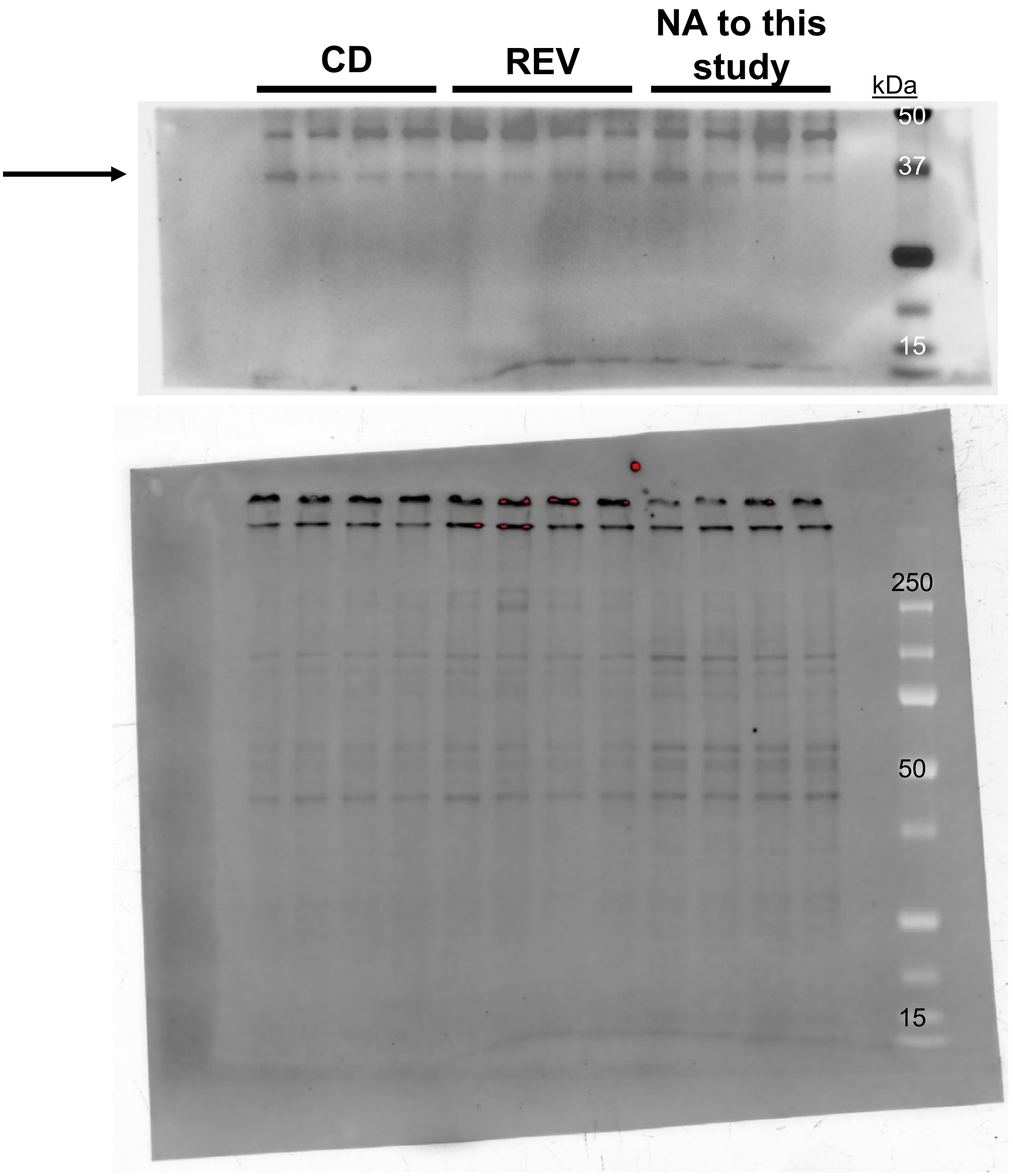

Supplemental Figure 12B: t-eIF2α & total protein

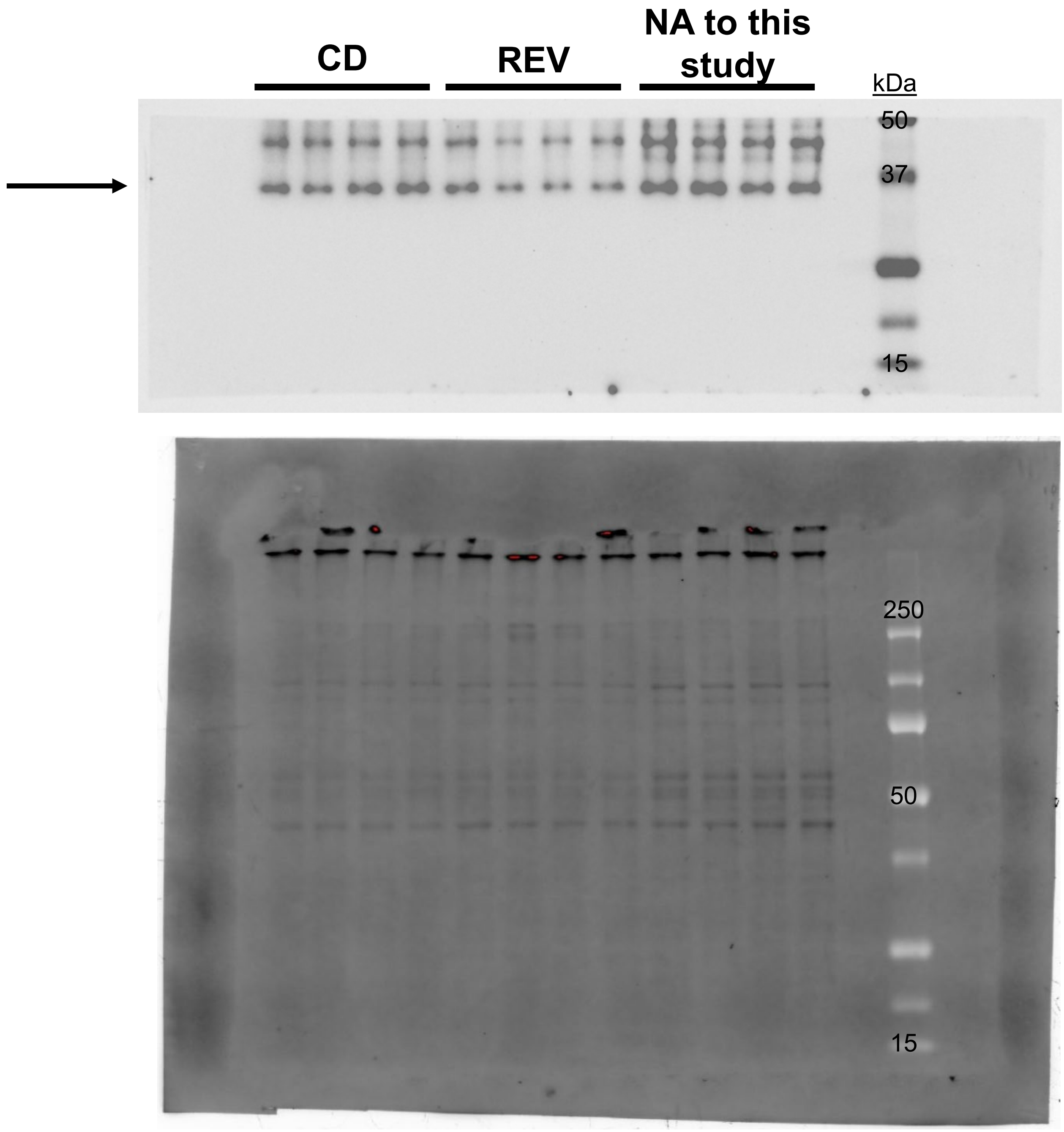

Supplemental Figure 12C: PDI & total protein

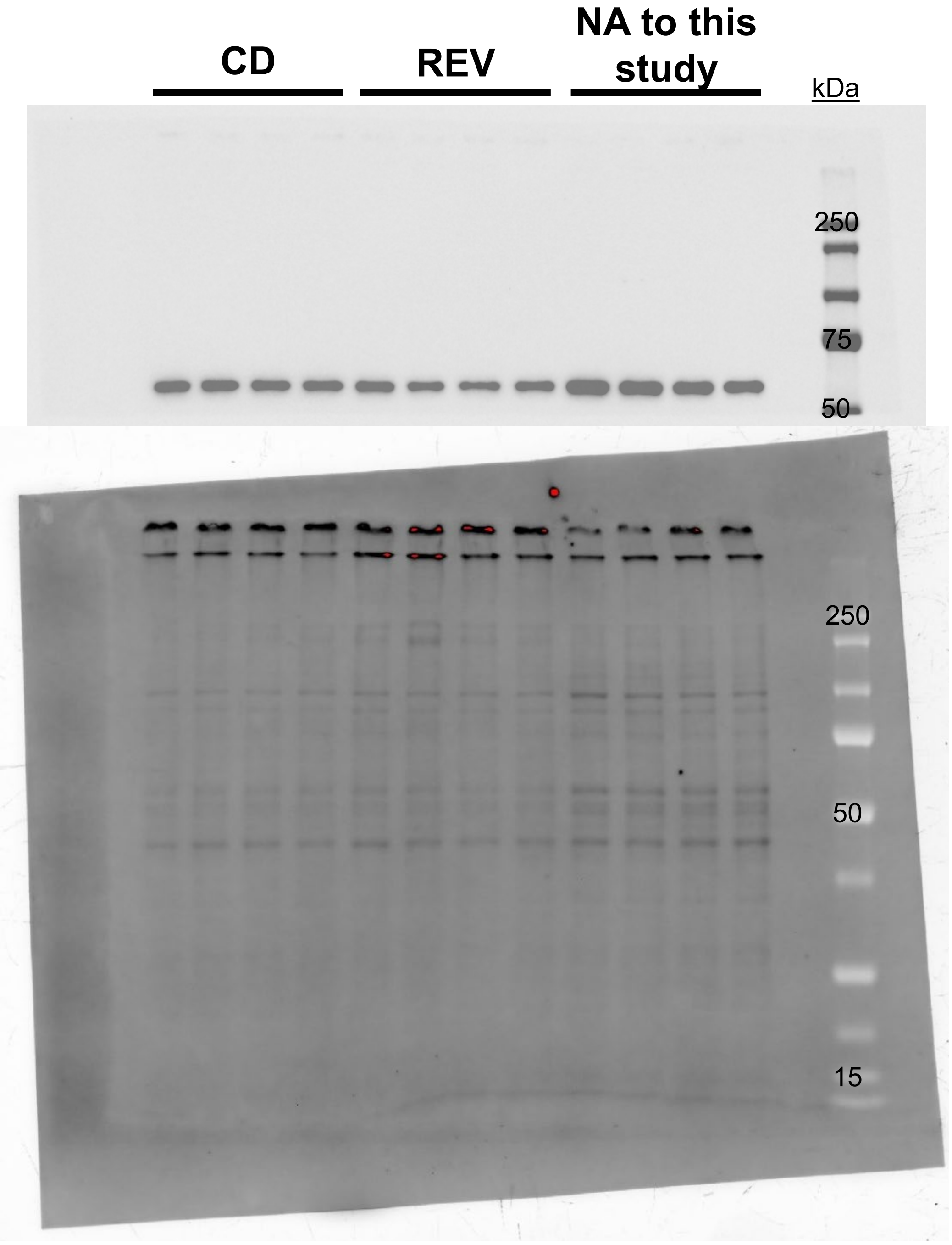

Supplemental Figure 12D: Ero1-Lα & total protein

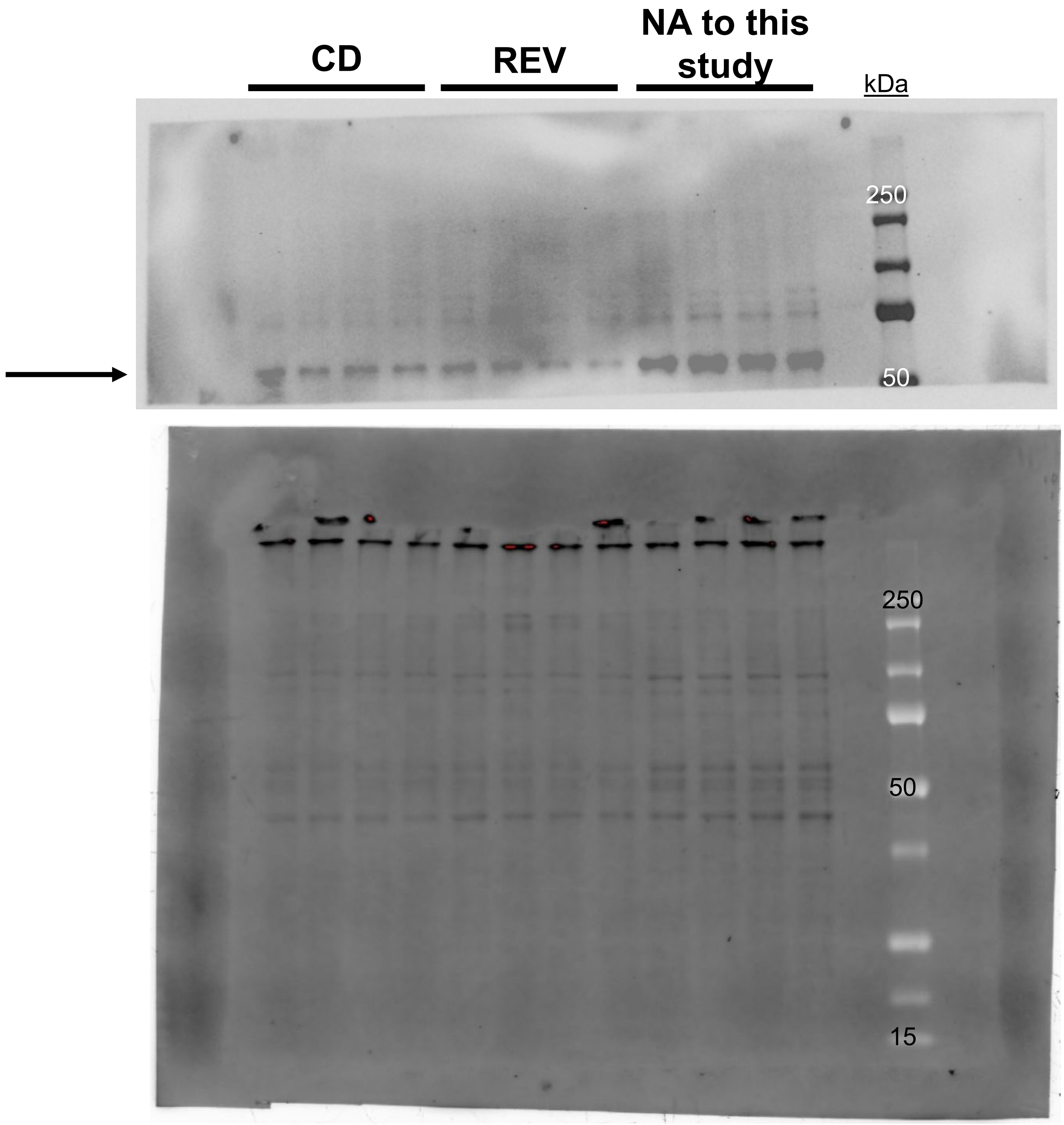

Supplemental Figure 12E: NIS & total protein

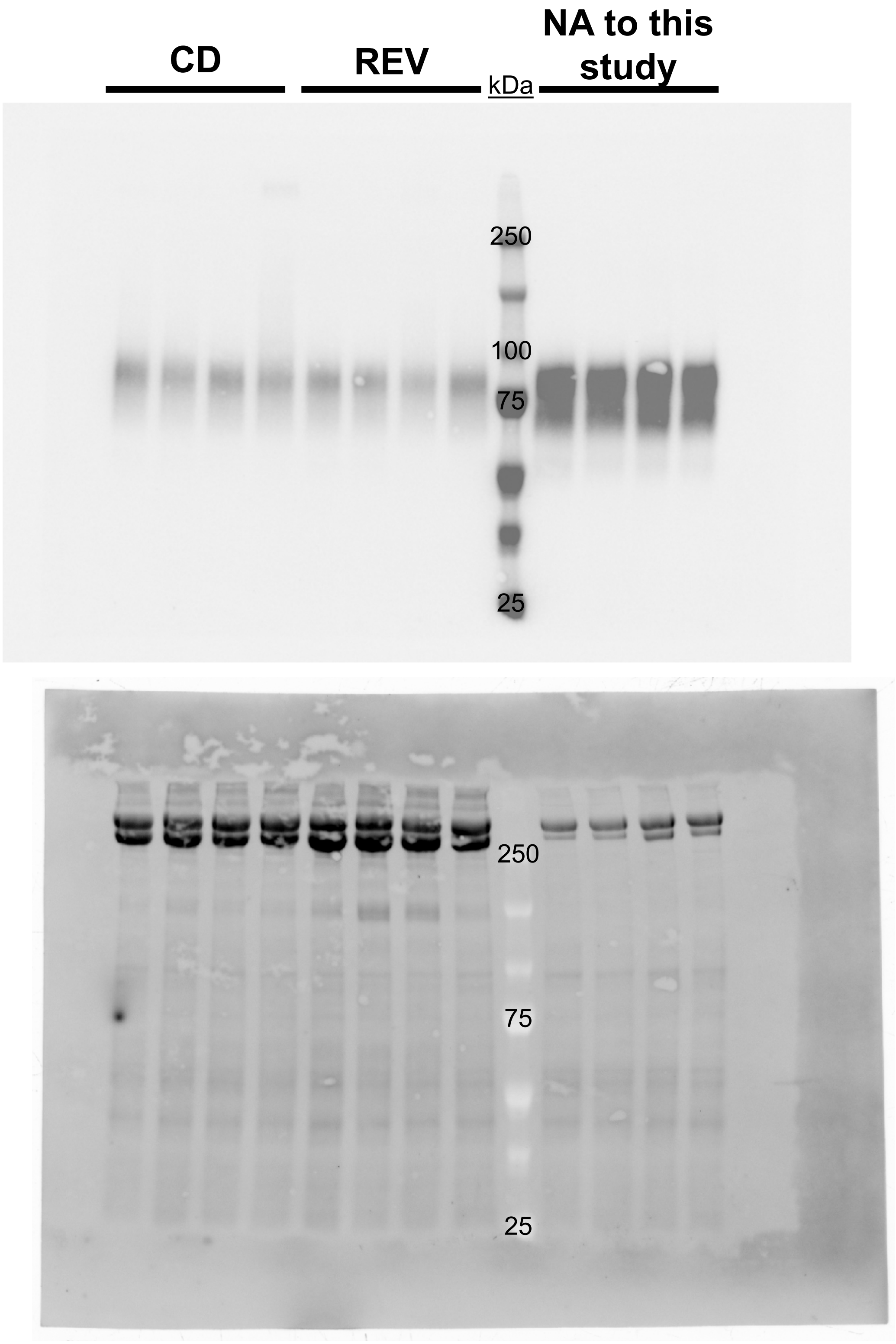

Supplement: Unedited blot and gel images [file jci-136-194207-s216.pdf]
